# Expression of CD44 by rhabdomyosarcoma: a new prognostic marker?

**DOI:** 10.1038/sj.bjc.6690442

**Published:** 1999-05

**Authors:** G Humphrey, D L Hazel, K MacLennan, I Lewis

**Affiliations:** Department of Paediatric Oncology and ICRF Cancer Medicine Research Unit, St James's University Hospital, Beckett Street, Leeds, LS9 7TF, UK

**Keywords:** CD44, rhabdomyosarcoma, prognosis

## Abstract

The expression pattern of CD44 standard and variant isoforms are prognostically significant in a number of malignancies. The aim of this study was to evaluate the role of the standard isoform of CD44 in predicting the clinical behaviour of rhabdomyosarcoma. Immunohistochemical analysis of CD44 was undertaken using a panel of antibodies recognizing the three core domains of the CD44 molecule. Labelling was repeated in triplicate and reported blind with respect to histological type and outcome. Tumours were characterized as positive in more than 60% of tumour cells labelled and negative if less than 40% of tumour cells labelled. Tumours with 40–60% of tumour cells labelling were considered indeterminate. Eleven of 20 favourable histology tumours were positive for CD44 compared with one of seven unfavourable tumours (*P* = 0.07). Eleven of 12 patients with CD44-positive tumours are alive in first remission compared with five of 15 CD44-negative tumours (*P* = 0.001). Expression of CD44 correlates directly with prognosis; however, larger studies are required so that multivariate analysis can be undertaken. © 1999 Cancer Research Campaign


*British Journal of Cancer* (1999) **80**(5/6), 918–921
© 1999 Cancer Research Campaign
Article no. bjoc.1998.0442

### Expression of CD44 by rhabdomyosarcoma: a new prognostic marker?

**G Humphrey[1], DL Hazel[1], K MacLennan[3] and I Lewis[1]**

[1]Department of Paediatric Oncology and [2]ICRF Cancer Medicine Research Unit, St James's University Hospital, Beckett Street, Leeds LS9 7TF, UK

**Summary** The expression pattern of CD44 standard and variant isoforms are prognostically significant in a number of malignancies. The aim of this study was to evaluate the role of the standard isoform of CD44 in predicting the clinical behaviour of rhabdomyosarcoma. Immunohistochemical analysis of CD44 was undertaken using a panel of antibodies recognizing the three core domains of the CD44 molecule. Labelling was repeated in triplicate and reported blind with respect to histological type and outcome. Tumours were characterized as positive in more than 60% of tumour cells labelled and negative if less than 40% of tumour cells labelled. Tumours with 40–60% of tumour cells labelling were considered indeterminate. Eleven of 20 favourable histology tumours were positive for CD44 compared with one of seven unfavourable tumours ($P = 0.07$). Eleven of 12 patients with CD44-positive tumours are alive in first remission compared with five of 15 CD44-negative tumours ($P = 0.001$). Expression of CD44 correlates directly with prognosis; however, larger studies are required so that multivariate analysis can be undertaken.

**Keywords:** CD44; rhabdomyosarcoma; prognosis

CD44 is an integral membrane glycoprotein which plays an important role in cell–substrate and cell–cell interactions including lymphocyte homing; lymphocyte, endothelial and mucosal interactions; cytokine release, T-cell activation; homotypic and heterotypic cell–cell adhesion and cytoskeletal interactions with the extracellular matrix (Lesley et al, 1993). Altered expression of CD44 has been observed in a large number of tumours of adult life (Matsumura and Tarin, 1992; Abbasu et al, 1993; Heider et al, 1993*a*; 1993*b*; Joensuu et al, 1993; Tanabe et al, 1993; Matsumura et al, 1994; Penno et al, 1994; Southgate et al, 1995; Harwood et al, 1996; Nagabhushan et al, 1996) and in the paediatric malignancy neuroblastoma (Favrot et al, 1993; Gross et al, 1994, 1995; Shtivelmann and Bishop, 1991). The aim of this study was to document the pattern of expression of CD44 by rhabdomyosarcomas and to determine the relationship of CD44 expression to prognosis.

### MATERIALS AND METHODS

Tumour samples collected prospectively at the time of diagnostic biopsy or definitive tumour excision in Leeds were used for this study. Tumour samples from 28 patients (age range 22 months to 15 years) diagnosed as having rhabdomyosarcoma between 1977 and 1994 were studied. Eleven patients were male and 17 female. Seventeen patients are alive in first remission, two are alive in second or subsequent remission, seven children are dead of disease and two children have died from treatment related complications. Distribution by site, stage and histological type are summarized in Table 1. Tumours had been characterized using an antibody panel including desmin, vimentin and MyoD1.

*Received 30 October 1997*
*Revised 8 April 1998*
*Accepted 14 April 1998*

*Correspondence to:* G Humphrey

Immunohistochemical analysis of CD44 was carried out using a standard streptavidin–biotin–horseradish peroxidase immunohistochemistry protocol [Streptavidin/ABC/HRP detection kit (DAKO)]. Previously characterized primary antibodies recognizing core epitope one (BRIC 222 at 1/60), epitope two (BRIC235 at 1/40) and epitope three (KZ-1 at 1/40) were obtained from International Blood Group Reference Laboratory, Bristol (Anstee et al, 1991). The secondary antibody was a biotinylated $F(ab')_2$ rabbit anti-mouse immunoglobulin (DAKO, Buckinghamshire, UK). Labelling was detected using diaminobenzidine chromagen and sections counterstained with haematoxylin. Fresh-frozen and paraffin sections were labelled in a similar fashion although formalin-fixed tissue was pretreated by dewaxing and microwave antigen retrieval (Gown et al, 1993). Control samples throughout included omission of primary antibody and the presence of non-tumour tissue within each section examined. Slides were assessed blind by an experienced tumour pathologist (KM) and labelling scored. Tumours were considered negative when tumour cell labelling was less than 40%, indeterminate when 40–60% of tumour cells labelled and positive when greater than 60% of tumour cells labelled.

Fisher's exact test with one-tail analysis of CD44 expression by histological subtype was undertaken. For analysis of disease progression the log-rank test was used for testing the difference between the groups. The level of confidence was set at $P = 0.05$. Statistical analysis was undertaken using SPSS for Windows (version 6.1) (Chicago, IL, USA).

### RESULTS

Identical labelling patterns were seen when matched pairs of fresh-frozen and formalin-fixed paraffin-embedded tumour samples were analysed. There was good correlation in the expression patterns obtained with labelling using BRIC222 and 235. The antibody KZ-1 labelled more cells in each sample than BRIC222 and 235.

**Table 1** Numbers of patients by site and stage of tumour

| | I | | II | | III | | IV | | Total |
|---|---|---|---|---|---|---|---|---|---|
| | F | U | F | U | F | U | F | U | |
| Orbit | 1 | | | | | | | | 1 |
| PMHN | | | | | | | 1 | | 1 |
| non-PMHN | | | | 1 | 1 | 2 | 1 | 1 | 6 |
| GU–BP | | | | | 6 | | 1 | | 7 |
| GU–non-BP | | | 1 | | 1 | | | | 2 |
| Extremity | 1 | 1 | | | 4 | | | | 6 |
| Other | | | | | 2 | 2 | | 1 | 5 |
| Total | 2 | 1 | 1 | 1 | 14 | 4 | 3 | 2 | 28 |

F = botryoid, embryonal, mixed embryonal undifferentiated and spindle cell tumours; U = alveolar and undifferentiated tumours. Stage I, II, III and IV refer to IRS clinical groups. PMHN = tumours involving parameningeal sites; non-PMHN = tumours of head and neck not involving parameningeal areas or orbit; GU–BP = tumours arising in bladder or prostate; GU–non-BP = tumours arising in genitourinary tract other than bladder or prostate; Extremity = tumour arising in a limb; Other = tumours arising in all other sites.

**Table 2** Table for testing probability of CD44 expression correlating with histological subtype

| | CD44-positive | CD44-negative | Total |
|---|---|---|---|
| Favourable | 11 | 9 | 20 |
| Unfavourable | 1 | 6 | 7 |
| Total | 12 | 15 | 27 |

Favourable = tumours of botryoid, embryonal or spindle cell type; unfavourable = alveolar and undifferentiated. $P = 0.07$.

**Table 3** Table for testing probability of association of CD44 expression with disease progression

| | CD44-positive | CD44-negative | Total |
|---|---|---|---|
| Disease-free in 1st remission | 11 | 5 | 16 |
| Disease progression | 1 | 10 | 11 |
| Total | 12 | 15 | 27 |

$P = 0.001$.

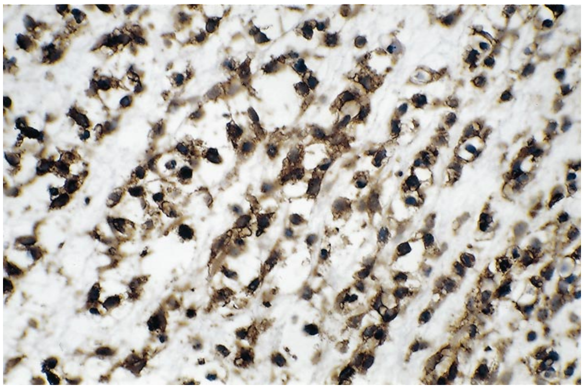

**Figure 1** Rhabdomyosarcoma with focal labelling of the majority of tumour cells for CD44, x120

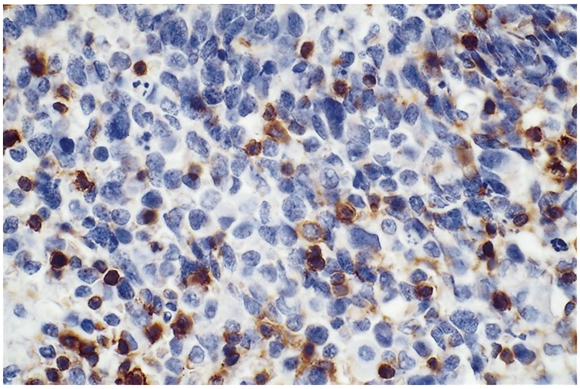

**Figure 2** Labelling of inflammatory cells for CD44 within an alveolar RMS negative for CD44, x120

One tumour had approximately equal numbers of labelled and unlabelled cells and has therefore not been included in statistical analysis by predominant cell phenotype. Eleven of 20 favourable histology tumours expressed CD44 (Figure 1) compared with one of seven unfavourable histology tumours (Figure 2) $P = 0.07$ (Table 2). Eleven of 12 patients with CD44 positive tumours are disease-free in first remission compared with five of 15 patients with CD44-negative tumours $P < 0.001$ (Table 3). The Kaplan–Meier survival curve is shown in Figure 3. Nineteen of 27 patients are alive more than 3 years from diagnosis. Seven of the remaining patients died from disease within 3 years of diagnosis and one patient died disease-free from treatment-related toxicity.

### DISCUSSION

CD44 is a transmembrane glycoprotein expressed on virtually all cell types where it acts as a receptor for hyaluronate (Picker et al, 1989; Culty et al, 1990; Lesley et al, 1993). It is encoded by a gene occupying 60–80 kb located at chromosome 11p13 and consists of at least 21 exons (Forsberg et al, 1989; Jackson et al, 1992; Screaton et al, 1992). The CD44 molecule has three core epitopes encoded by ten exons with alternative mRNA splicing of the remaining exons generating multiple isoforms (CD44v). The standard form of CD44 (CD44s) is expressed on almost all cell types and is heavily glycosylated. Variant isoforms are expressed in a cell- and tissue-specific manner (Arch et al, 1992; Herrlich et al, 1993; Lesley et al, 1993; Mackay et al, 1994).

© Cancer Research Campaign 1999

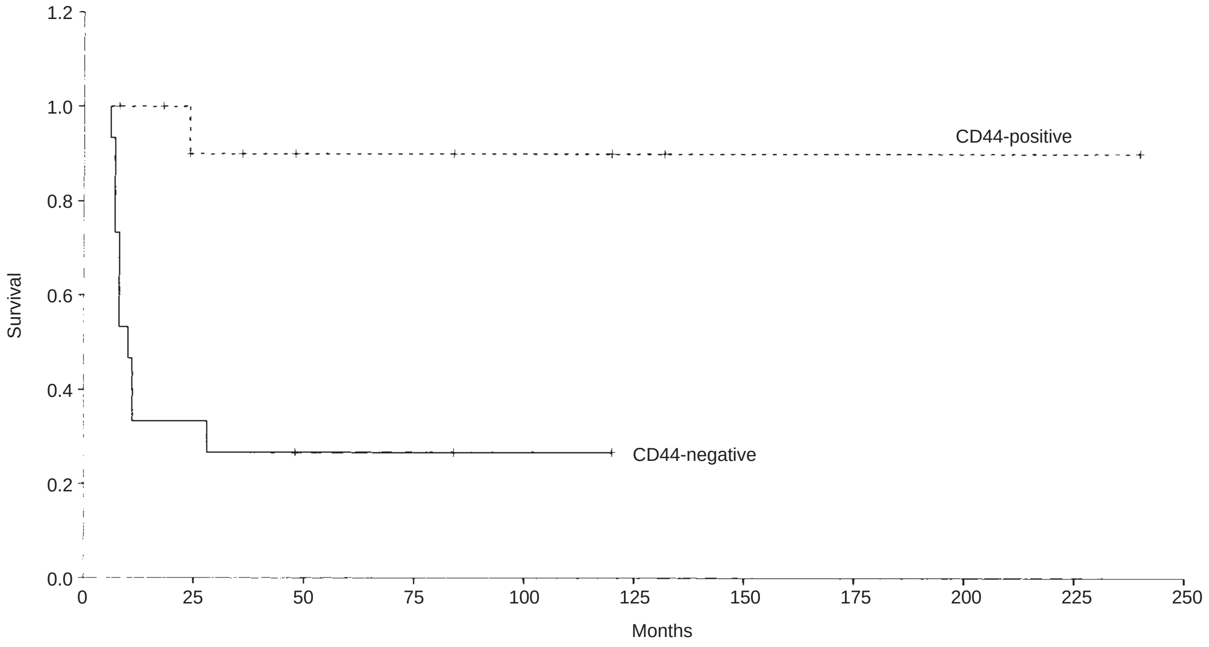

**Figure 3** Kaplan–Meier survival curve demonstrating improved survival in patients with CD44-positive tumours, *P* = 0.001

Qualitative and quantitative changes in expression of CD44 have been demonstrated in vitro in the vascular dissemination of melanoma (Birch et al, 1991) and lymphoma cells (Sy et al, 1991), and in the migration of rat pancreas carcinoma cells on the extra-cellular matrix (Günthert et al, 1991). In vivo, enhanced or up-regulation of CD44 (core or variant) expression has been found to be related to tumour progression in breast (Joensuu et al, 1993), colorectal (Abbasu et al, 1993*a*; Tanabe et al, 1993; Wielenga et al, 1993), gastric (Heider et al, 1993*a*; Mayer et al, 1993), cervical (Dall et al, 1994) and bladder (Matsumura et al, 1994; Southgate et al, 1995) carcinomas, non-Hodgkin’s lymphoma (Koopman et al, 1993) and brain tumours (Terpe et al, 1993). Conversely, loss of or reduction in expression of CD44v isoforms is associated with disease progression in squamous cell (Salmi et al, 1993) and endometrial carcinomas (Fujita et al, 1994). In addition, loss of CD44s isoforms has been reported in metastatic prostatic (Nagabhushan et al, 1996) and bladder cancer (Southgate et al, 1995) and melanomas during their vertical growth phase (Harwood et al, 1996).

Several studies have confirmed the potential importance of CD44 as a prognostic indicator for neuroblastoma tumours with stage 1–3 and 4s disease expressing CD44 (Favrot et al, 1993; Gross et al, 1994, 1995; Christiansen et al, 1995; Terpe et al, 1995). Studies of stage 4 neuroblastomas comparing MYCN amplification with CD44 expression have shown that there is a highly significant inverse relationship between MYCN amplification and CD44 expression (Favrot et al, 1993; Gross et al, 1994, 1995; Christiansen et al, 1995; Terpe et al, 1995). In rhabdomyosarcoma, MYCN amplification appears to be a relatively infrequent observation except in those of alveolar subtype (Dias et al, 1990; Mailet et al, 1992; Driman et al, 1994; Tsuda et al, 1998). This study combined with that of Saxon et al (1997) suggests that most alveolar rhabdomyosarcomas are CD44-negative; therefore, studies correlating CD44 expression with MYCN amplification should be carried out on embryonal and alveolar rhabdomyosarcomas to see if MYCN amplification is directly related to histological type and to establish if the two are causally related.

In 1958, Horn and Enterline divided rhabdomyoscarcomas into four subtypes – embryonal, botryoid, alveolar and pleiomorphic. In an attempt to improve the prognostic value of basic histology an alternative classification divides tumours into: favourable (botryoid and spindle cell), moderately favourable (all other embryonal tumours), and unfavourable (solid alveolar, alveolar and undifferentiated) (Newton et al, 1995). In 1997, Saxon reported that none of five alveolar rhabdomyosarcomas studied expressed CD44 and that embryonal tumours had a heterogenous pattern of labelling but did not attempt to correlate this with disease outcome. This study suggests that alveolar tumours are predominantly CD44-negative. This small study appears to indicate that low expression of CD44 correlates with poor outcome, these results must be interpreted with caution because of the small sample size and possible confounding influence of subset analysis. To confirm the hypothesis that low CD44 expression predicts poor outcome and to establish if this is independent of histological subtype further studies should be performed using a large retrospective data set with multivariate analysis.

### REFERENCES

Abbasu AM, Chester KA, Talbot IC, Macpherson AS, Boxer G, Forbes A, Malcolm A and Begent R (1993) CD44 is associated with proliferation in normal and neoplastic human colorectal epithelial cells. *Eur J Cancer* **14**: 1995–2002

Anstee DJ, Gardner B, Spring FA, Holmes CH, Simpson KL, Parson SF, Mallinson G, Yousaf SM and Judson PA (1991) New monoclonal antibodies to CD44 and CD58: their use to quantify CD44 and CD58 on normal human erythrocytes and to compare the distribution of CD44 and CD58 in human tissues. *Immunology* **74**: 197–205

Arch R, Wirth K, Hofmann M, Ponta H, Matzuka S, Herrlich P and Zoller M (1992) Participation in normal immune responses of a metastasis-inducing splice variant of CD44. *Science* **257**: 682–685

*© Cancer Research Campaign 1999*

Birch M, Mitchell S and Hart IR (1991) Isolation and characterization of human melanoma cell variants expressing high and low levels of CD44. *Cancer Res* **51**: 6660–6667

Christiansen H, Sahin K, Berthod F, Hero B, Terpe H-J and Lampert F (1995) Comparison of DNA aneuploidy, chromosome 1 abnormalities, MYCN amplification and CD44 expression as prognostic factors in neuroblastoma. *Eur J Cancer* **31A**: 541–544

Culty M, Miyake K, Kincade PW, Silorski E, Butcher EC and Underhill C (1990) The hyaluronate receptor is a member of the CD44 (H-CAM) family of cell surface glycoproteins. *J Cell Biol* **111**: 2765–2774

Dall P, Heider KH, Hekele A, van Minckwitz G, Kaufmann M, Ponta H and Herrlich P (1994) Surface protein expression and messenger RNA-splicing analysis of CD44 in uterine cervical cancer and normal cervical epithelium. *Cancer Res* **54**: 3337–3341

Dias P, Kumar P, Marsden HB, Gattamaneni HR, Heighway J and Kumar S (1990) N-*myc* gene is amplified in alveolar RMS but not embryonal RMS. *Int J Cancer* **45**: 593–596

Driman D, Thorner PS, Greenberg ML, Chilton-MacNeill S and Squire J (1994) MYCN gene amplification in rhabdomyoscarcoma (RMS). *Cancer* **73**: 2231–2237

Favrot MC, Combaret V and Lasset C (1993) CD44 – a new prognostic marker for neuroblastoma. *N Engl J Med* **329**: 1965

Forsberg UH, Ala-Kapee MM, Jalkanen S, Andersson LC and Schroder J (1989) The gene for human lymphocyte homing receptor is located on chromosome 11. *Eur J Immunol* **19**: 409–412

Fujita N, Yaegashi N, Ide Y, Sato S, Nakamura M, Ishiwata I and Yajima A (1994) Expression of CD44 in normal human versus tumor endometrial tissues: possible implication of reduced expression of CD44 in lymph-vascular space involvement of cancer cells. *Cancer Res* **54**: 3922–3928

Gown AM, de Wever N and Battifora H (1993) Microwave-based antigenic unmasking. A revolutionary new technique for routine immunohistochemistry. *Appl Immunohistochem* **1**: 256–266

Gross N, Beretta C, Peruisseau G, Jackson D, Simmons D and Beck D (1994) CD44H expression by human neuroblastoma cells: relation to MYCN amplification and lineage differentiation. *Cancer Res* **54**: 4238–4242

Gross N, Beck D, Beretta C, Jackson D and Perrusisseau G (1995) CD44 expression and modulation on human neuroblastoma tumours and cell lines. *Eur J Cancer* **31A**: 471–475

Günthert U, Hofmann M, Ruddy W, Reber S, Zoller M, Haussmann I, Matzku S, Wenzel A, Ponta H and Herrlich P (1991) A new variant of glycoprotein CD44 confers metastatic potential to rat carcinoma cells. *Cell* **65**: 13–24

Harwood CA, Green MA and Cook MG (1996) CD44 expression in melanocytic lesions: a marker of malignant progression? *Br J Dermatol* **135**: 876–882

Heider K-H, Dammrich J, Skroch-Angel P, Müller-Hermelink H-K, Vollmers P, Herrlich P and Ponta H (1993*a*) Differential expression of CD44 splice variants in intestinal- and diffuse-type human gastric carcinomas and normal gastric mucosa. *Cancer Res* **53**: 4197–4203

Heider K-H, Hofmann M, Hors E, van den Berg F, Ponta H, Herrlich P and Pals ST (1993*b*) A human homologue of the rat metastasis-associated variant of CD44 is expressed in colorectal carcinomas and adenomatous polyps. *J Cell Biol* **120**: 227–233

Herrlich P, Zoller M, Pals ST and Ponta H (1993) CD44 splice variant: metastases meet lymphocytes. *Immunol Today* **14**: 395–399

Lesley J, Hyman R and Kincade PW (1993) CD44 and its interaction with extracellular matrix. *Adv Immunol* **54**: 271–335

Jackson DG, Buckley J and Bell JI (1992) Multiple variants of the human lymphocyte homing receptor CD44 generated by insertions at a single site in the extracellular domain. *J Biol Chem* **267**: 4732–4739

Joensuu H, Klemi PJ, Toikkannen S and Jalkanen S (1993) Glycoprotein CD44 expression and its association with survival in breast cancer. *Am J Pathol* **143**: 867–874

Koopman G, Heider KH, Horst E, Adolf GR, van den Berg F, Ponta H, Herrlich P and Pals ST (1993) Activated human lymphocytes and aggressive non-Hodgkin's lymphomas express a homologue of rat metastasis-associated variant of CD44. *J Exp Med* **177**: 897–904

Mackay CR, Terpe H-J, Stauder R, Marston WL, Stark H and Günthert U (1994) Expression and modulation of CD44 variant isoforms in humans. *J Cell Biol* **124**: 71–82

Mailet MW, Robinson R and Burgart LJ (1992) Genomic alterations in sarcomas: a histologic correlative study with use of oncogene panels. *Mod Pathol* **5**: 410–414

Matsumura Y and Tarin D (1992) Significance of CD44 gene products for cancer diagnosis and disease evaluation. *Lancet* **340**: 1053–1058

Matsumura Y, Hanbury D, Smith J and Tarin D (1994) Non invasive detection of malignancy by identification of unusual CD44 gene activity in exfoliated cancer cells. *Br Med J* **308**: 619–624

Mayer B, Jauch KW, Günthert U, Figdor CG, Schilberg FW, Funke I and Johnson JP (1993) De novo expression of CD44 and survival in gastric cancer. *Lancet* **342**: 1019–1022

Nagabhushan M, Pretlow TG, Guo Y-J, Amini SB, Pretlow T and Sy M-S (1996) Altered expression of CD44 in human prostate cancer during progression. *Am J Clin Pathol* **106**: 647–651

Newton WA Jr, Gehan EA, Webber BL, Marsden HB, Van Unnik AJ, Hamoudi AB, Tsokos MG, Shimada H, Harms D, Schmidt D, Ninfo V, Cavazzano AO, Gonzalez-Crussi F, Parham DM, Reiman HH, Asmar L, Beltangady MS, Sachs NE, Triche TJ and Maurer HM (1995) Classification of rhabdomyosarcomas and related sarcoms. pathological aspects and proposals for a new classification – an Intergroup Rhabdomyosarcoma Study. *Cancer* **76**: 1073–1085

Penno MB, August JT, Baylin ST, Mabry M, Linnala I, Lee VS, Croteau D, Yang XL and Rosada C (1994) Expression of CD44 in human lung tumors. *Cancer Res* **54**: 1381–1387

Picker LJ, Nakache M and Butcher EC (1989) Monoclonal antibodies to the human lymphocyte homing receptors define a novel class of adhesion molecules on diverse cell types. *J Cell Biology* **109**: 927–937

Salmi M, Grön-Virs K, Sointu P, Grenman R, Kalimo H and Jalkanen S (1993) Regulated expression of exon v6 containing isoforms of CD44 in man: down regulation during malignant transformation of tumors of squamocellular origin. *J Cell Biol* **122**: 432–442

Saxon BR, Byard RW and Han P (1997) Cellular expression of adhesion factors in childhood rhabdomyosarcoma. *Ped Pathol Lab Med* **17**: 259–266

Screaton GR, Bell MV, Jackson DG, Cornelis FB, Gerth U and Bell JI (1992) Genomic structure of DNA encoding the lymphocyte homing receptor CD44 reveals at least 12 alternatively spiced exons. *Proc Natl Acad Sci USA* **89**: 12160–12164

Shtivelmann E and Bishop JM (1991) Expression of CD44 is repressed in neuroblastoma cells. *Mol Cell Biol* **11**: 5446–5453

Southgate J, Tredjdosiewicz LK, Smith B and Selby PJ (1995) Patterns of splice variant and CD44 expression by normal human urothelium in situ and in vitro and by bladder-carcinoma cell lines. *Int J Cancer* **62**: 449–456

Sy MS, Guo YJ and Stamenkovic I (1991) Distinct effects of two CD44 isoforms on tumor growth in vivo. *J Exp Med* **174**: 859–866

Tanabe KK, Ellis LM and Saya H (1993) Expression of CD44R1 adhesion molecule in colon carcinomas and metastases. *Lancet* **341**: 725–726

Terpe H-J, Zimmer C, Günthert U and Korf B (1993) CD44-expression in human brain tumors. *Clin Neuropathol* **12**: 271–272

Terpe H-J, Christiansen H, Gonzalez M, Berthold F and Lampert F (1995) Differentiation and prognosis of neuroblastoma in correlation to the expression of CD44s. *Eur J Cancer* **31A**: 549–552

Tsuda H, Shimosato Y, Upton MP, Yokota J, Terada M, Ohira M, Sugimura T and Hiroshashi S (1988) Retrospective study on amplification of N-*myc* and c-*myc* genes in pediatric solid tumors and its association with prognosis and tumor differentiation. *Lab Invest* **59**: 321–327

Wielenga VJ, Heider K-H, Offerhaus AJ, Güther R, van den Berg FM, Ponta H, Herrlich P and Pals ST (1993) Expression of CD44 variant proteins in human colorectal cancer is related to tumor progression. *Cancer Res* **53**: 4754–4756

© Cancer Research Campaign 1999
